# Acquired *EGFR* L858R mutation following *ALK*-TKI resistance in lung adenocarcinoma: a case report

**DOI:** 10.3389/fonc.2026.1779992

**Published:** 2026-03-05

**Authors:** Wenying Peng, Ruying Duan, Runxiang Yang, Susu Qu, Mengyuan Dong, Ruofan Chen, Chunxiang Luo

**Affiliations:** 1 The Second Department of Oncology, The Third Affiliated Hospital of Kunming Medical University & Yunnan Cancer Hospital & Peking University Cancer Hospital Yunnan, Kunming, China; 2 The Second Department of Internal Medicine, Kunming Xishan District People’s Hospital, Kunming, Yunnan, China

**Keywords:** Anaplastic lymphoma kinase, case report, epidermal growth factor receptor, lung adenocarcinoma, targeted therapy, ALK-TKI resistance

## Abstract

Reports of secondary mutations in mutual exclusive driver genes after resistance to targeted therapy are rare. We present a patient with Anaplastic lymphoma kinase (*ALK*) fusion lung adenocarcinoma who received sequential treatment with ALK tyrosine kinase inhibitor (TKI) (crizotinib, PFS:32.3 months and then conteltinib, PFS: 29 months). Upon further disease progression, a lung biopsy and next-generation sequencing (NGS) revealed acquired secondary driver mutations including Epidermal Growth Factor Receptor (*EGFR)* L858R and *ALK* mutation of F1174L. Subsequently, the patient switched to third generation *EGFR*-TKI treatment with almonertinib. This case suggests *EGFR* mutation is one of the mechanisms of *ALK*-TKI resistance, highlights the value of re-biopsy in identifying potentially targetable resistance mechanisms and underscores the spatiotemporal heterogeneity of tumors under the selective pressure of *ALK*-TKI.

## 1 Introduction

The development of *ALK*-TKIs has significantly improved the survival and prognosis of patients with *ALK*-mutated non-small cell lung cancer (NSCLC), however, drug resistance and disease progression are inevitable. The resistance mechanism of *ALK*-TKIs includes primary resistance and acquired resistance, and acquired resistance can be further divided into ALK-dependent resistance, ALK-independent resistance and other mechanisms(such as phenotypic transformation (1)).The ALK-dependent resistance refers to mutations in the ALK tyrosine kinase domain (1), accounting for about 30-40% (2), such as the G1202R mutation (3), L1196M mutation (4); and ALK-independent resistance, accounting for about 50% (2), is mainly caused by activation of bypass pathways other than the target, such as activation of the EGFR (5)or MET pathway (6), etc. Besides, there are still many resistance mechanisms for *ALK*-TKI that have not been clarified. For *ALK*-TKI-resistant patients, re-biopsy and genetic testing are paramount in identifying resistance mechanisms and guiding treatment. In *ALK*-mutated NSCLC, the coexistence of *ALK* mutations and *EGFR* mutations leading to *ALK*-TKI resistance is rarely reported.

This case represents the first reported instance of a triple mutation profile—persistent *ALK* fusion, acquired *ALK* F1174L and *EGFR* L858R—following *ALK*-TKI resistance, which complements the current *ALK*-TKI resistance mechanism and indicates tumor heterogeneity posing significant challenges to treatment.

## 2 Case presentation

A 51-year-old male never-smoker without family history of cancer presented to the hospital due to a mass in his right lower limb. CT scan showed bone destruction of the right tibia with a mass formation, to further clarify the diagnosis, the patient underwent a right tibial biopsy. The results on August 6, 2019 indicated metastatic adenocarcinoma, most likely of pulmonary origin. A concurrent chest CT scan showed a patchy lesion in the middle lobe of the right lung, enlarged lymph nodes in the mediastinum and right hilar region. Consequently, an electronic bronchoscopy was performed: tumor infiltration was identified in the right middle lobe bronchus. On August 22, 2019, pathology from the brushing and smear further confirmed non-small cell carcinoma (NSCLC), thereby establishing the primary pulmonary lesion. Following orthopedic evaluation, he underwent tibial tumor resection and the immunohistochemistry supported a diagnosis of lung adenocarcinoma metastasis, and the genetic testing of the tibial resection specimen via a 10-gene Polymerase Chain Reaction (PCR) panel identified an *EML4-ALK* fusion with other driver genes reported wildtype (*EGFR*, *ROS1*, *RET*, *KRAS*, *BRAF*, *HER2*, *NRAS*, *PIK3CA*, and *c*-*MET*).

Diagnosed with right lung adenocarcinoma with tibial and hilar lymph node metastases, cT1N2M1, stage IV, *EML4-ALK* fusion. The patient started first-line crizotinib in November 2019, and achieved stable disease (SD) with a 32.3-month progression-free survival (PFS), until disease progression was confirmed on July 2022 for enlarged mediastinal lymph nodes. The patient declined further biopsy and genetic testing. On July 29, 2022, the second generation *ALK*-TKI conteltinib (CT-707) was initiated. From July 2022 to August 2024, the patient was regularly monitored with chest CT, brain MRI. Throughout this period, the best response was partial response (PR) and PFS was 29 months, all subsequent surveillance imaging (including the last scan in September 2024) indicated persistent PR, and the patient remained asymptomatic. New brain metastases were identified on regular imaging dated December 29, 2024, which accompanied with progression of the primary lung lesion, and new bone metastases. Notably, the patient was still asymptomatic at this point of radiographic progression. Based on RECIST 1.1 criteria, disease progression (PD) was assessed, with the best response during treatment being PR, and PFS was 29 months. After the patient’s disease progressed following CT-707 treatment and with the patient’s consent, we conducted a biopsy and gene sequencing on December 31, 2024 to guide subsequent treatment. Given CT-detected mediastinal lymph node enlargement, this biopsy employed endobronchial ultrasound-guided transbronchial needle aspiration (EBUS-TBNA). While performing a brush biopsy of the right middle lobe bronchus, targeted tissue samples were obtained from mediastinal group 4R lymph nodes. Pathology results showed the 4R mediastinal lymph node smear indicated adenocarcinoma. Concurrently, next-generation sequencing(NGS) using a 733-gene tissue panel of the 4R lymph node tissue sample obtained via EBUS-TBNA revealed an *EML4-ALK* fusion (abundance 31.22%), *ALK* F1174L kinase domain mutation (abundance 15.22%), and a *de novo* acquired *EGFR* L858R mutation (abundance 5.22%). He received whole-brain stereotactic radiotherapy (35 Gy/5 fractions/7 Gy) and third-generation Epidermal Growth Factor Receptor -TKI almonertinib. At each outpatient follow-up visit, the patient reported good adherence to the prescribed medication regimen and denied experiencing any significant adverse effects. However, a follow-up examination on February 11, 2025 revealed disease progression: cranial MRI and chest CT scans indicated enlargement of known intracranial metastases and the primary pulmonary lesion, and the known bone metastases also showed progression. The treatment strategy was better to change systemic medication. So, we switched to lorlatinib treatment (100mg once daily) on February 12, 2025. Unfortunately, due to financial constraints and complained of no symptom remission, the patient discontinued treatment and follow-up approximately one month later.

**Figure 1:**
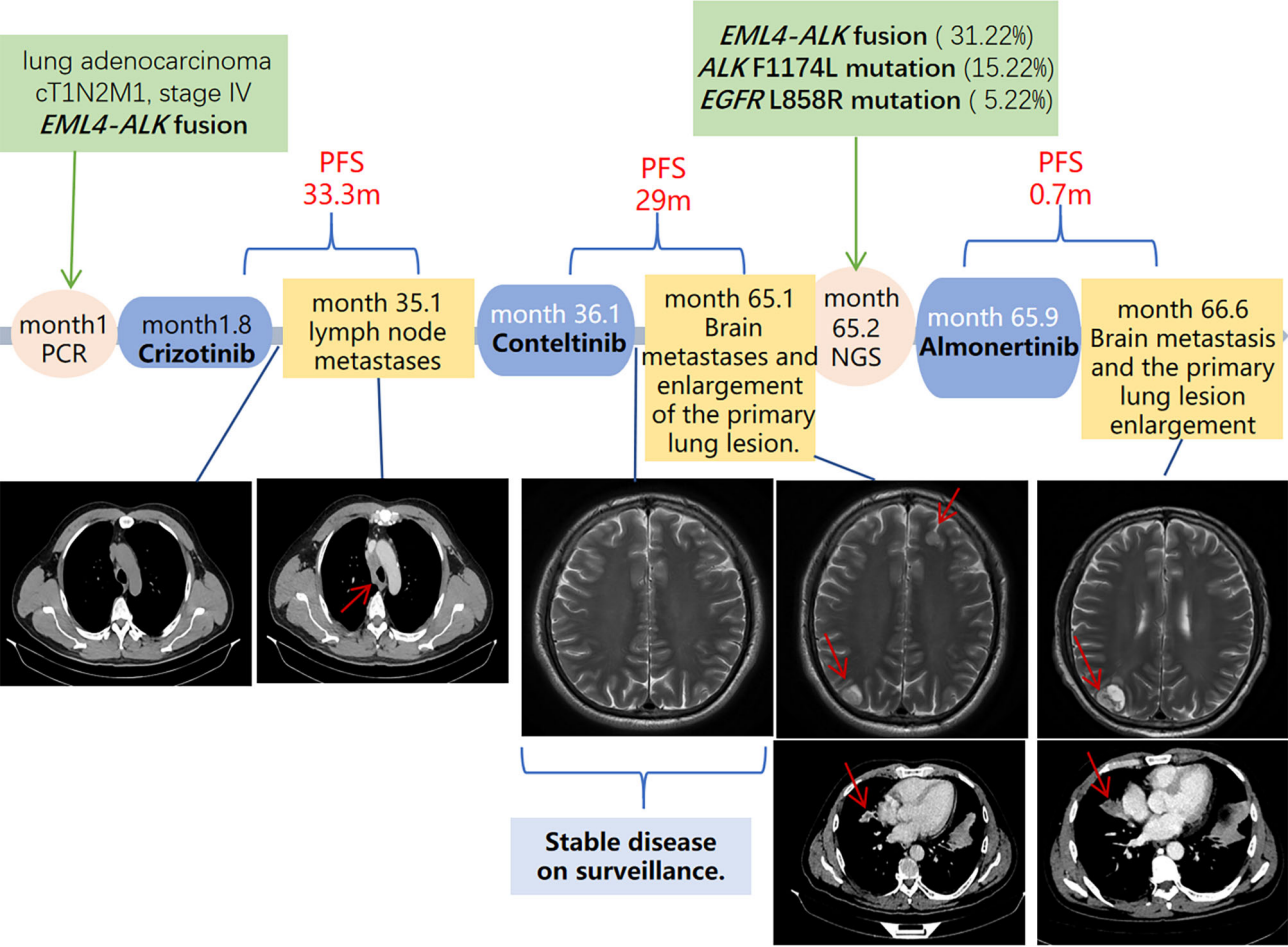
The timeline of treatment and disease progression. PCR, Polymerase Chain Reaction; NGS, Next-Generation Sequencing Technology; PFS, progression-free survival.

The patient’s clinical course, including sequential treatments, treatment responses, and key progression events, are summarized in **Figure 1**.

## 3 Discussion

Anaplastic lymphoma kinase (*ALK*) rearrangements occur in 3%–7% of non-small cell lung cancer (NSCLC) (7), conferring sensitivity to several *ALK*-TKIs approved so far: crizotinib, alectinib, brigatinib, ceritinib, lorlatinib, ensartinib, iruplinalkib and envonalkib etc. Crizotinib significantly improves patient survival compared to chemotherapy as first-line treatment (8), but resistance remains an unsolved challenge. *ALK*-TKI resistance mechanisms are categorized as ALK-dependent resistance; ALK-independent resistance and other mechanisms (Histologic transformation, Drug efflux pump, etc.) (2). Although *EGFR* and *ALK* mutations are often mutually exclusive at diagnosis (9, 10), previous case reported acquired *ALK*-fusion may be a potential mechanism of resistance to *EGFR*-TKIs *(*11), and this case suggests that *EGFR* driver mutations may also be one of the mechanisms of acquired resistance to *ALK*-TKIs.

Multiple studies indicated that EGFR phosphorylation was elevated in crizotinib-resistant cell lines, even in *EGFR* wild-type cells (12). Dardaei et al. noted that *ALK*-TKI treatment can trigger compensatory activation of the EGFR-RAS-MAPK axis, promoting resistance (13). We speculate that the emergence of the *EGFR* L858R mutation subclone in this case is one of the mechanisms of drug resistance, resulting from tumor evolution under sustained selective pressure from ALK-TKI (crizotinib followed by conteltinib). The *EGFR* L858R mutant cell subclone gains a survival advantage by activating the downstream RAS-MAPK pathway, ultimately forming a distinct adaptive escape clone.

Besides this case, another case report has presented an *ALK*-positive lung adenocarcinoma patient who acquired an *EGFR* L858R mutation after progressing on crizotinib and brigatinib (14). Notably, upon resistance, the original *ALK* rearrangement was lost in both liquid and tissue biopsies. Therefore, whether this *EGFR* mutation was a secondary alteration of the original tumor or a *de novo* event in an independent second primary cancer warrants further discussion. In contrast, the re-biopsy sample of our case revealed two new mutations indicative of possible resistance mechanisms: an *ALK* acquired F1174L mutation and an *EGFR* L858R mutation, providing clear evidence of profound tumor clonal heterogeneity.

A subsequent biopsy upon disease progression after 29 months of response revealed an *ALK* F1174L mutation (15.22%). The *ALK* F1174L mutation is commonly observed as a primary driver mutation in neuroblastoma, located at the C-terminal end of the ALK kinase domain αC helix. In NSCLC, it can enhance ATP binding and constitutively activate ALK, making it a resistance mutation to crizotinib (15) and ceritinib (16). Following crizotinib resistance, the patient received conteltinib (CT-707), a locally developed second-generation *ALK*-TKI. conteltinib overcomes multiple crizotinib-resistant mutations in the ALK kinase domain by targeting the FAK/PDPK1/AKT1 pathway (17). In preclinical studies, conteltinib is more potent than crizotinib against mutant kinases(CT‐707 IC50 = 3.8 nM; crizotinib IC50 = 15.9 nM) (18),it can inhibit various Crizotinib-resistant mutations including F1174L in the ALK kinase domain (19), its clinical efficacy against NSCLC harboring *ALK-*F1174L warrants further investigation, as evidenced by the eventual progression in this case, and the biopsy at the time of this progression also revealed an *EGFR* L858R mutation, combined with a history of treatment, suggesting that F1174L is likely the mechanism leading to crizotinib resistance, while the subsequent emergence of *EGFR* L858R may be the mechanism leading to CT-707 resistance. Furthermore, this case represents the first reported instance of a triple mutation profile—persistent *ALK* fusion, acquired *ALK* F1174L, and *EGFR* L858R—following *ALK*-TKI resistance, suggesting the possibility of identifying novel resistance mechanisms and heterogeneity through re-biopsy after targeted therapy resistance.

In this case the initial ARMS-PCR test could not detect F1174L. Performing NGS on tissue or circulating tumor DNA (ctDNA) before and after crizotinib could clarify its evolutionary trajectory, but this was not feasible in this case due to unforeseen circumstances. In practice, re-biopsy is often limited by invasiveness and sample availability. Liquid biopsy via ctDNA analysis can circumvent these challenges by providing real-time monitoring of resistance dynamics, thereby addressing the limitations of this study.

Based on the patient’s NGS results following disease progression on CT-707 (*EML4-ALK* fusion, abundance 31.22%; an *ALK* F1174L kinase domain mutation, abundance 15.22%; an acquired *EGFR* L858R mutation, abundance 5.22%), posing significant challenges for subsequent treatment,and the patient’s biopsy results demonstrated persistent *ALK*-dominant tumor driver signaling. Selecting an *ALK* inhibitor would theoretically be the optimal choice.

Although lorlatinib has demonstrated efficacy against the F1174L mutation (20, 21), and ALK signaling remains predominant, our final decision prioritized the *EGFR*-TKI almonertinib combined with whole-brain radiotherapy. This decision was based on multidimensional considerations. Firstly, the emergence of *EGFR* L858R following progression on CT-707 represents a certain newly acquired mutation and distinct bypass resistance mechanism. Lorlatinib is not expected to inhibit the newly acquired *EGFR* L858R bypass resistance mutation, concurrently, the patient developed multiple new brain metastases despite previously stable disease on all prior follow-ups, indicating comprehensive disease progression. So, we decided to prioritize the potent *EGFR*-TKI almonertinib (22) to inhibit this newly emerging clone, combined with intracranial radiotherapy thereby controlling the progression of intracranial metastatic lesions. Simultaneously, although the patient exhibits high *ALK* mutation abundance, prior exposure to multiple *ALK*-TKI regimens (crizotinib, CT-707) raises concerns about overlapping resistance mechanisms upon reintroduction of *ALK* inhibitors (23). Since there are no standard treatment guidelines for patients with dual or multiple mutations, and final decisions depend on clinical consideration, full discussion and patient’s individualized preference. While studies suggest dual-targeted therapy may overcome targeted resistance in NSCLC patients (24), there are still risks of overlapping adverse reactions and patient financial constraints. More clinical case reports are helpful for further validation. However, we indeed gave patient several choices: 1) Combination targeted therapy with *EGFR*-TKI and *ALK*-TKI but adverse effect might be heavy, 2) sequential alternately use of *EGFR*-TKI and *ALK*-TKI, 3) Monotherapy of *EGFR*-TKI or third generation *ALK*-TKI lorlatinib, 4) Chemotherapy with bevacizumab, pemetrexed, and platinum (cisplatin/carboplatin). Due to several reasons, the patient ultimately chose to sequential use of the two TKIs, and the cheaper one (almonertinb) was given earlier. After disease progression following almonertinib therapy, the patient then switched to *ALK*-TKI treatment lorlatinib. Unfortunately, the patient did not come back to hospital to receive further follow-up and examinations after approximately one month dose of medication.

This case showed that tumor heterogeneity exhibits multiple evolutionary trajectories and endows tumor with robust proliferation and invasiveness, which increases difficulties in monitoring resistance and impairs the efficacy of precision therapy, leading to rapid disease progression and a poor prognosis.

## 4 Conclusions

We reported a case of patient with *ALK* fusion lung adenocarcinoma, following two lines of *ALK*-TKIs treatment resistance, the genetic testing showed an *ALK* fusion, *ALK* F1174L mutation and *EGFR* L858R mutation. This suggests *EGFR* L858R is one of the mechanisms of *ALK*-TKI resistance and highlights the critical role of re-biopsy upon progression to identify resistance mechanisms and profound tumor heterogeneity, which can severely compromise precision therapy.

## Data Availability

The datasets presented in this article are not readily available because these anonymized mutation results are included within the article. The raw sequencing datasets are not publicly available due to patient privacy and confidentiality restrictions, as they are considered part of the patient’s clinical record. Requests to access the datasets should be directed to luochunxiang@kmmu.edu.cn.
